# Exposure to Occupational Hazards among Health Care Workers in Low- and Middle-Income Countries: A Scoping Review

**DOI:** 10.3390/ijerph18052603

**Published:** 2021-03-05

**Authors:** Rajni Rai, Sonia El-Zaemey, Nidup Dorji, Bir Doj Rai, Lin Fritschi

**Affiliations:** 1 School of Public Health, Curtin University, Bentley, WA 6102, Australia; Sonia.El-Zaemey@curtin.edu.au (S.E.-Z.); Lin.Fritschi@curtin.edu.au (L.F.); 2 Faculty of Nursing and Public Health, Khesar Gyalpo University of Medical Sciences of Bhutan, Thimphu 11001, Bhutan; nidupdorji.dorji@gmail.com; 3 Regional Livestock Development Centre Wangduephodrang, Wangduephodrang 14001, Bhutan; birdoj_rai@yahoo.com

**Keywords:** occupational hazards, health care workers, low- and middle-income countries

## Abstract

Health care workers are exposed to numerous workplace hazards. The implementation of safety measures in high-income countries has largely mitigated these risks. However, in many low- and middle- income countries (LMICs), resources to institute safety measures are lacking, increasing the risk of occupational exposures to these hazards. The aim of this scoping review is to map and synthesize the available research on occupational hazards among health care workers in LMICs, identify research gaps and inform policy. Searches for relevant articles were conducted in five electronic databases using a broad range of search terms. The inclusion criteria were: quantitative observational or experimental studies which examined exposure to one or more occupational hazards among health care workers in a LMCI; and the article was published in English in a peer-reviewed journal. A total of 99 studies met the inclusion criteria, and data were extracted from these studies. Large proportions of health care workers in LMICs were exposed to biological hazards (bloodborne pathogens, tuberculosis), psychosocial hazards (workplace violence, burnout, job dissatisfaction), ergonomic hazards (musculoskeletal complaints), and chemical hazards (exposure to latex and antineoplastic drugs). The implementation of risk reduction strategies was suboptimal. The majority of the literature was on biological hazards (48%), and research on other hazards was limited in comparison. Occupational safety needs to become a priority public health issue to protect health care workers in LMICs. More research is needed to understand the magnitude of the problem in these countries.

## 1. Introduction

Health care workers are at potential risk of harm from exposure to numerous hazardous agents encountered in their workplace [1]. The most recent and visible example is the ongoing COVID-19 pandemic, which has showcased the vulnerability of health care workers and demonstrated the importance of ensuring their safety [2].

In addition to exposures to emerging diseases, health care workers are routinely exposed to other infectious agents such as tuberculosis, influenza, HIV, and Hepatitis B, which have been the primary focus of research and safety programs [3]. Health care workers are also exposed to various chemical hazards and agents that have been linked to long-term adverse health effects. Chemicals used in health care settings such as ethylene oxide, formaldehyde, and antineoplastic drugs have been linked to cancers and adverse reproductive outcomes [4,5,6]. Exposure to latex and cleaning and disinfecting agents has been associated with occupational asthma among health care workers [7,8]. Musculoskeletal disorders and injuries, and various psychosocial hazards such as workplace violence, stress, and burnout are other well-recognised occupational hazards among health care workers [9,10,11].

Recognising these risks, safety measures and standards to protect health care workers have been instituted in high-income countries and have largely succeeded in mitigating these hazards [12]. However, in many low- and middle-income countries (LMICs), occupational health and safety is often neglected [13]. These deficiencies in occupational health have been attributed to a lack of political commitment, insufficient resources, poor data collection systems, and weak enforcement of regulations. Occupational health research has shown that providing a safe work environment increases organizational commitment and worker retention [14]. Poor working conditions and threats to health have been reported to contribute to problems in recruitment and retention of health care workers in LMICs, augmenting the issue of health care worker shortages in these countries [15].

In order to institute any prevention and safety intervention, it is important to understand the magnitude of the problem. The majority of the literature on occupational hazards in health care workers has originated in high-income countries, and research from LMICs on this topic is reported to be limited [16]. Findings from studies conducted in high-income countries cannot be generalised to LMICs because exposures in LMICs are likely to be different from high-income countries due to differences in legislation and regulations, health care systems, work practices and the availability of control measures. There is a need to determine the scope and volume of available research conducted on this topic in LMICs and to identify any research gaps. Apart from a narrative literature review conducted in 2016, which was limited in scope and included only 46 studies, there are no other reviews available on this topic [16].

Scoping reviews have been described by Arksey and O’Malley as those which “aim to map rapidly the key concepts underpinning a research area and the main sources and types of evidence available, and can be undertaken as standalone projects in their own right, especially where an area is complex or has not been reviewed comprehensively before” [17]. A revised definition of scoping reviews was proposed by Daudt et al. as “scoping studies aim to map the literature on a particular topic or research area and provide an opportunity to identify key concepts, gaps in literature; and types and sources of evidence to inform practice, policymaking, and research” [18]. Therefore, a scoping review was conducted to map and synthesize the available research on exposure to occupational hazards among health care workers in LMICs, to identify any research gaps and to inform policy to improve the safety of health care workers.

## 2. Methods

This review was conducted according to the methodological framework for scoping reviews outlined by Arksey and O’Malley [17], Levac et al. [19], Colquhan et al. [20], and The Joanna Briggs Institute [21]. It is reported in accordance with the Preferred Reporting Items for Systematic reviews and Meta-Analyses extension for Scoping Reviews (PRISMA-ScR) [22]. It was guided by the research question ‘What is known from the existing literature about exposure to occupational hazards among health care workers in LMICs?’

### 2.1. Search Strategy

The key terms relating to the research question were identified as follows: ‘health care workers’, ‘health workers’, ‘health personnel’, ‘health professionals’, ‘nurses’, ‘doctors’, ‘laboratory workers’ ‘occupational hazards’, ‘occupational risks’, ‘occupational diseases’, ‘occupational health’, ‘occupational injuries’, ‘occupational accidents’, ‘low-and-middle-income countries’, ‘low-income countries’, and ‘developing countries’. The search strategy was developed by the research team in consultation with an academic librarian. Using these key terms and their associated mapped subject headings and MeSH terms, searches were conducted in the electronic databases MEDLINE, Scopus, CINAHL, Embase, and PsycINFO till 1 May 2020 (Table S1. Search strategy for Medline (Ovid) (date of search: 1 May 2020)). Original peer-reviewed articles in the English language were the only limits applied to the searches to maintain a breadth of coverage. Bibliographies of the included studies were also checked to ensure that all relevant studies had been included in the review. Grey literature was not included.

### 2.2. Study Selection

Studies were selected based on the following inclusion criteria: (1) participants were health care workers as classified by the World Health Organization (WHO) [23], (2) the study was conducted in a low- and middle- (both lower- and upper-) income country as classified by the World Bank classification of countries, 2020 [24], (3) the study topic was on exposure to occupational hazards, (4) the type of study was a quantitative observational or experimental study, and (5) the article was published in English in a peer-reviewed journal. Studies were excluded if they were qualitative in design, case series or case reports, reviews, conference presentations or dissertations. The only exception to the application of the selection criteria was on studies on tuberculosis. For tuberculosis, since a systematic review on tuberculosis among health care workers in LMICs had been published in 2006 [25], only studies conducted after this period on this topic were included. Studies on night shift work were also excluded.

After removing duplicates, one reviewer (RR) assessed the articles by titles and abstracts and applied the inclusion and exclusion criteria to select the full-text articles to be retrieved. Any uncertainties related to study selection at this stage was discussed with the research team till a consensus was reached. Full-text articles were then screened independently by two reviewers (RR and SE-Z) to finalize their inclusion in the review. Any disagreement regarding the determination of study inclusion in the review at this stage was resolved by consulting a third reviewer (LF). Manual searches of the reference lists of included studies were also conducted.

### 2.3. Charting of the Data

Data were extracted from the studies and charted on a table by one reviewer (RR). This included author, year of publication, country of study origin, aims, study population and sample size, study design and methodology, and key findings. A second reviewer (LF) then extracted data from ten randomly selected studies using the data charting form to ensure that the data extraction approach was consistent with the research question and study aims.

### 2.4. Collating and Summarising the Results

The study characteristics, which included the year of publication, study design and methodology, location, participant characteristics, the topic researched, and the study outcomes, were first tabulated. This was performed to provide a descriptive numerical summary of the studies included in the review. A thematic analysis was then carried out, and the studies were sorted into occupational hazards groups based on the WHO classification of occupational hazards in health care workers [26]. These two steps assisted in identifying the dominant areas of research, their location and methodology and any research gaps. The findings are then described as a narrative review.

## 3. Results

**Figure 1:**
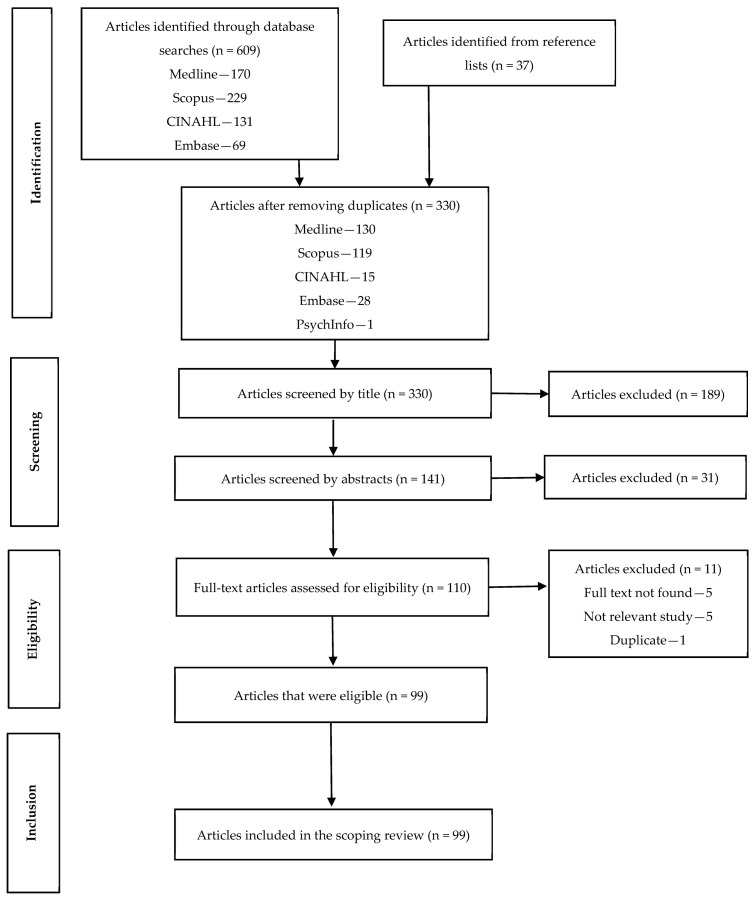
Flow chart illustrating the scoping review study selection process.

The database searches identified 609 articles, with a further 37 articles identified from a search of reference lists (Figure 1). After removing duplicates, 330 articles were screened by titles followed by abstract examinations of 141 articles. The review of abstracts resulted in 110 articles for full-text examination, of which 99 articles met the inclusion criteria and were included in the review.

**Figure 2:**
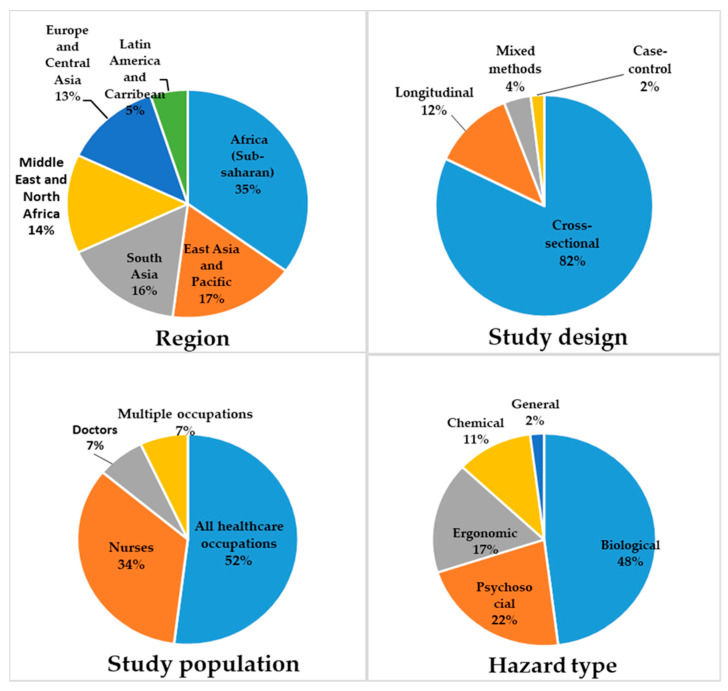
Characteristics of studies.

**Figure 3:**
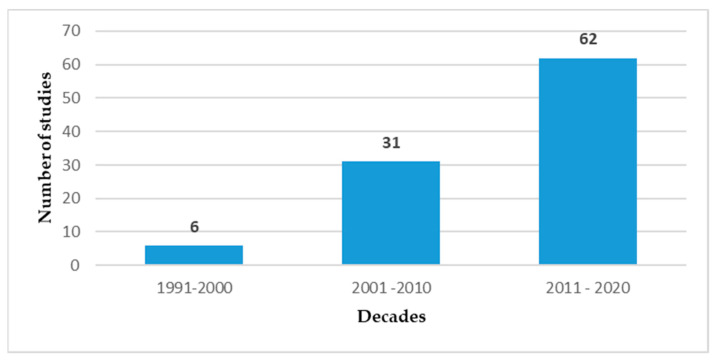
Number of studies by a decade of publication.

The majority of the studies (34 of 99) were conducted in the Sub-Saharan African region (according to the World Bank regions), were cross-sectional in design (82), and participants were all health care workers (51) (Figure 2). Fifty one studies were conducted in district/state hospitals and primary care centres, and 48 were conducted in tertiary care centres. The included studies were published after 1991, with six studies published in the 1990s, 31 studies published between 2001 and 2010, and 62 studies between 2011 and 2020 (Figure 3).

Almost half the studies (47) were on biological hazards, 22 studies were on psychosocial hazards, 17 were on ergonomic hazards, and 11 were on chemical hazards (Figure 2). In addition, there were two studies that investigated the different types of occupational hazards in general. Among the studies on biological hazards, the majority (38/47) examined exposure to bloodborne pathogens and nine studies (after 2006) examined exposure to tuberculosis (Table 1). Among the studies on psychosocial hazards, 12 studies examined workplace violence and safety climate, six studies examined the prevalence of burnout and its risk factors and four studies examined work environment and job satisfaction (Table 2). The studies on ergonomic hazards mainly investigated the prevalence of musculoskeletal complaints and their risk factors (Table 3). Among the studies on chemical hazards, six studies examined exposure to latex, and five examined exposure to antineoplastic drugs (Table 4).

## 4. Discussion

This study aimed to map and synthesize the available research on occupational hazards among health care workers in LMICs. The research conducted on this topic is quite substantial as evidenced by the 99 articles included in this review. However, half of these studies were on biological hazards, and research on the other types of hazards was minimal in comparison. The findings of this review also show that research on occupational hazards in LMICs has increased considerably in the last decade, perhaps indicating an increasing recognition of occupational health and safety of health care workers in these countries.

### 4.1. Biological Hazards

#### 4.1.1. Bloodborne Pathogens

The majority of the literature on biological hazards was on the occupational transmission of bloodborne pathogens, such as Hepatitis B, HIV, and Hepatitis C, through needlestick/sharps injuries and splash accidents. Health care workers from LMICs are at increased risk of transmission of bloodborne pathogens because of the high population prevalence of these diseases and the fact that safety measures to reduce these risks are inadequate [126].

The prevalence of needlestick injuries was variably reported in the studies included in this review, with some studies reporting prevalence in the past year, some over the entire career and a few reporting it in the past 3 months, 6 months and 5 years. The prevalence of needlestick injuries in the past year was reported in 12 studies and showed a wide variation, ranging from 27% in a study conducted in Nigeria to 82% in a study conducted in China [28,33,35,37,40,43,46,47,48,58,63,64]. The prevalence of needlestick injuries over the entire career was reported in nine studies and ranged from 32.4% in a study conducted in Ethiopia to 86.2% in a study from China [35,37,43,44,54,61,62,63,64]. The incidence of needlestick injuries was reported in two studies. A study conducted in Kenya reported an incidence rate of 0.97 needlestick injuries per health care worker per year [42] and a study from Turkey reported an incidence of 2.18 exposures/person-years [59].

Needlestick injuries were more common than accidental splashes [38,45,55,57,58], and syringes caused most of the needlestick injuries [43,45,48,51]. The highest frequencies of injuries were reported by nurses, doctors (mainly surgeons and interns), dental personnel, and cleaners [27,28,31,32,43,45,46,48,51,55,57,59,61,63]. The risk factors for injuries were lack of training, heavy workloads, long working hours, not using gloves, recapping of needles, and using syringes frequently [37,43,48,51,59].

Various risk reduction strategies have been recommended to decrease occupational exposures to bloodborne pathogens, such as the use of standard precautions, vaccination against Hepatitis B, and post-exposure prophylaxis (PEP) for Hepatitis B and HIV [127]. Compliance with standard precautions for infection control was suboptimal as reported in a number of studies from various countries [29,30,32,33,36,38,47,48,49,50,52,54,58,60,64,124,125]. Barriers to compliance reported were shortage of equipment, inadequate staffing, and lack of training [39]. Unsafe injection practices such as recapping of needles and reusing syringes were also prevalent [28,34,49,50,53,54]. Most of the needlestick injuries were not reported and treated [33,44,56,58]. There were seven studies that reported on Hepatitis B vaccination status. The vaccination status (completed 3 doses of vaccine) was low in most of the studies ranging from 8% to 56.1% [34,41,46,51,54,60], except for a study conducted in China (71%) [33]. Among all health care workers, vaccination rates were lowest in housekeeping personnel [34,46]. There were only three studies that examined post-exposure prophylaxis for HIV and these studies reported a low uptake of PEP by health care workers and that almost half of those who started PEP discontinued the treatment due to side effects of the drugs [27,32,42]. There were no studies reporting the use of HBV immunoglobulin for post-exposure prophylaxis for HBV infection, which could be due to its unavailability in LMICs [34].

Taken together, the findings of this review show that needlestick and splash injuries are prevalent in LMICs and risk reduction strategies to protect health care workers from these infections are suboptimal.

#### 4.1.2. Tuberculosis

A systematic review on tuberculosis among health care workers in LMICs published in 2006 reported a high occupational risk of tuberculosis, with a latent tuberculosis infection (LTBI) prevalence of 54% (range 33% to 79%), an incidence of 0.5% to 14.3% per year, and an attributable risk due to nosocomial exposure from 25 to 5361 per 100,000 per year [25]. As with transmission of bloodborne infections, health care workers in LMIC are at an increased risk of exposure to tuberculosis due to high population tuberculosis rates and limited resources to institute control practices [128]. As compared to high-income countries where there are strict infection control practices to protect health care workers, even basic infection control strategies to reduce transmission in health care facilities in LMICs are lacking and tuberculosis control is mainly focused on case detection and treatment [128,129].

This present review included studies conducted after 2006, and found that occupational tuberculosis transmission is still a significant problem in LMICs. The prevalence of LTBI as reported by five studies in this review ranged from 23.6% to 76.5% when assessed using interferon-gamma release assays (IGRAs), and from 59.1% to 97.6% when assessed with tuberculin skin tests (TSTs) [65,67,68,70,73]. IGRAs are newer tests that use antigens that are more specific and hence are less likely to be affected by previous BCG vaccination status and non-tuberculosis mycobacterial infection, which are the drawbacks of TSTs [129]. In the systematic review, only one study had used IGRAs to detect LTBI prevalence. There was one study in this present review that reported the incidence rates of LTBI test conversion, a prospective study conducted in Georgia from 2009 to 2011, which reported conversion rates of 17.1 per 100 person-years for TST and 22.8 per 100 person years for IGRAs [68].

There were four studies examining active tuberculosis among health care workers in this review [66,69,71,72]. A study conducted in India reported a pulmonary tuberculosis incidence rate of 314 per 100,000 person-years among health care workers and that this was 1.86 times higher than that of the general population [66]. Another study conducted in South Africa reported a tuberculosis incidence rate of 1985 per 100,000 person-years among health care workers, which was double the incidence of tuberculosis in the general population [69]. A study conducted in China used low-dose lung CT examinations to detect active tuberculosis, and reported that the incidence and prevalence rates of active tuberculosis in health care workers were >2.8 times and >4.1 times greater than that of the general population, respectively [72].

The risk factors for acquiring tuberculosis identified in this review were working in high-risk areas (tuberculosis facilities/wards, medical wards, outpatient departments, microbiology laboratories, radiology departments), belonging to certain occupation groups (nurses, microbiology laboratory technicians, and radiology technicians), working for >10 years, increasing age, and having co-morbidities such as diabetes and HIV [65,66,67,68,69,70,71,72,73].

In summary, the prevalence and incidence of LTBI in health care workers in LMICs is very high and active tuberculosis among health care workers is approximately two times higher than that of the general population.

### 4.2. Psychosocial Hazards

#### 4.2.1. Workplace Violence

The majority of studies on psychosocial hazards in this review were on workplace violence. Workplace violence has been reported as a significant problem in the health care sector throughout the world [10]. In this review, the prevalence of experiencing some form of violence in the workplace was high and ranged from 60.8% to 82.2% [76,78,82]. The prevalence varied depending on the specific type of violence measured (e.g., physical, verbal, sexual). Verbal abuse was the most common type of violence experienced by health care workers, with a prevalence ranging from 30.5% to 95.9% [75,76,77,78,79,80,81,83,84,85]. The prevalence of physical abuse ranged from 2.3% to 36.8% [75,76,78,79,80,81,83,84,85] and that of sexual harassment ranged from 0.7% to 21.8% [75,76,83,84]. Patients and their families were the most commonly reported perpetrators of verbal and physical abuse, while co-workers and patients were the most commonly reported perpetrators of sexual harassment [74,75,76,77,78,79,81,85]. The risk factors for workplace violence identified in this review were working in certain high-risk areas (out-patient departments, emergency departments, operation theatres and in-patient clinics), lower safety climate levels at work, working in shifts, having heavy workloads and younger age [74,75,77,81,84].

Being a victim of workplace violence can result in a range of negative consequences (psychological, physical, emotional, social, work functioning, quality of care, and financial) [130]. Five studies included in this review reported on the consequences and associations of workplace violence [74,78,80,82,85]. Three studies reported on psychological consequences, where exposure to workplace violence was associated with anxiety, depressive symptoms and major depression, and burnout [80,82,85]. Two studies reported on work functioning consequences and found that almost half (42.9% and 45%) of the participants who experienced workplace violence reported a decline in work productivity [74,78].

#### 4.2.2. Burnout

Burnout, as described by Maslach et al. [131], comprises of three dimensions: emotional exhaustion, depersonalization, and low personal accomplishment. Health care workers are known to be at an increased risk of burnout due to the inherent nature of their job which exposes them to high levels of emotional and psychological stress [11]. Burnout has been found to be associated with absenteeism, high turnover rates, low morale, and decrease in the quality of care.

Four of the six studies included in this review examined burnout among doctors (residents and anaesthesiologists) [86,88,90,91] and two studies examined it in acute and critical care nurses [87,89]. These studies reported a high prevalence of burnout. The prevalence of high levels of burnout in at least one dimension ranged from 51.3% to 80% [86,88,91]. Emotional exhaustion, depersonalization, and low personal accomplishment prevalence ranged from 39.4% to 67.7%, 38% to 68.4%, and 23.8% to 50.3%, respectively [86,88,90,91]. The work-related risk factors for burnout identified in this review were long working hours, experiencing a major stress at work, not having the right team to work with, lack of autonomy at work, and negative psychosocial work environments (as measured by perceived effort-reward imbalance). Personal risk factors were reported by only two studies and these included female gender, being single and having children [86,87]. Only one study reported on the consequences of burnout and this study found that burnout was independently associated with decreased adherence to infection control practices [89].

#### 4.2.3. Work Environment and Job Satisfaction

Two studies included in this review examined job satisfaction and work environment among nurses and reported that more than fifty percent of the nurses (56.4% to 67.1%) were not satisfied with their jobs and only 31% perceived their work environment to be of high quality [93,95]. Advancement in the job, recognition, work security and a good work environment were the factors that were reported to be positively associated with job satisfaction. One study examining nurses’ satisfaction with night shift work reported that only 43% of these nurses were satisfied with their night shifts, and the factors associated with the low levels of satisfaction were inadequate staffing and inadequate equipment for protection from hazards [94]. A longitudinal study conducted in China examined psychosocial work environment and intention to leave among nurses and reported a 16.3% prevalence of intention to leave and an incidence rate of 14.5% [92]. Increased emotional demands, decreased workplace commitment, decreased meaning of work and decreased job satisfaction were the factors reported to be associated with intention to leave.

The delivery of quality health care depends largely on the quality of staff delivering these services [132]. Satisfied workers are known to be more efficient and productive, thus contributing to the provision of better quality services. Job dissatisfaction, on the other hand, is associated with absenteeism and higher employee turnover rates. Providing a good work environment is a key factor in improving employee job satisfaction, organizational commitment and intention to remain [14].

In summary, the prevalence of verbal and physical abuse, and burnout were reported as being extremely high in these studies. In addition, satisfaction with work was low. These factors impact on retention of health care workers which is particularly important in the context of LMICs since these countries already face a shortage of health care workers [133].

### 4.3. Ergonomic Hazards

Musculoskeletal disorders are a common cause for work-related disability and absenteeism, resulting in substantial financial consequences in the form of workers’ compensation and medical expenditure [134]. Health care worker are at an increased risk of musculoskeletal disorders and there is an extensive body of literature from high-income countries examining these disorders among different occupation groups within the health care sector (nurses, surgeons, physical therapists, dentists) [9,135,136,137].

The studies on ergonomic hazards included in this review examined prevalence and risk factors of musculoskeletal disorders among health care workers. Thirteen studies examined musculoskeletal disorders using the Nordic Musculoskeletal Questionnaire, mainly among nurses (10/13 studies) [96,97,98,101,102,103,104,106,107,108,111,112], and four studies examined only low-back pain [99,100,109,110]. The prevalence of musculoskeletal complaints in at least one body site in the past twelve months was reported in 12 studies and ranged from 50.7% to 95%. The most commonly reported body site for these complaints was the lower back (35.3% to 78.2%). The prevalence reported for the other regions of the body ranged from 28% to 49.8% for the neck, 23.5% to 52.1% for the shoulders, 20.7% to 54% for the upper back and 11% to 68.7% for the knees. There was only one study that examined work-related injuries, in which 38.6% of the nurses in the study reported experiencing at least one work-related injury in the past twelve months [99].

The occupational physical risk factors for musculoskeletal complaints identified in this review were working in the same position for prolonged periods, working in a bent or twisted position, lifting and transferring patients, handling many patients, and performing repetitive tasks [102,106,112]. The occupational psychosocial risk factors for musculoskeletal complaints identified were high levels of stress, anxiety, mental exhaustion, limited support in the workplace, low decision latitude, increased workload, monotonous work, job inexperience, and absenteeism [96,98,101,104,108,111,112].

In summary, there were few studies of musculoskeletal disorders among LMIC health care workers, and they found a very high prevalence of musculoskeletal complaints in at least one body site. There was a lack of studies on work-related injuries.

### 4.4. Chemical Hazards

The studies on chemical hazards in this review mainly examined exposure to latex and latex allergy. Three studies examined the prevalence of latex allergy symptoms among health care workers and reported a prevalence ranging from 16% to 18% [114,115,117]. The occupational risk factors for latex allergy reported in these studies were the number of years using latex gloves, using latex gloves for >1 h per day, using >15 pairs of powdered gloves per day, longer duration of working as a health care worker, using chlorhexidine and working as an operation theatre nurse. Two studies conducted in Turkey and Thailand examining the prevalence of latex sensitization by measuring latex-specific IgE antibody levels reported a prevalence of 4.2% and 4.4%, respectively, and that the prevalence was higher in hospitals where gloves with higher protein levels were used [118,119].

The use of less allergenic alternatives such as powder-free latex gloves and nitrile gloves has been recommended to control latex exposures among health care workers [12]. A study conducted in South Africa examined the prevalence of latex allergy and sensitization after the introduction of hypoallergenic powder-free and lightly powdered latex gloves [116]. The prevalence of latex allergy and sensitization reported in this study was 5.9% and 7.1%, respectively. The authors concluded that health care workers using hypoallergenic powder-free latex gloves were at risk of developing latex sensitization and recommended that a cost-effective alternative that eliminated latex from the health care environment was required in resource poor countries.

Five studies included in this review examined exposure to antineoplastic drugs, mainly safe handling practices, and reported that adherence to control measures was suboptimal. A study conducted in Egypt reported a lack of medical surveillance programs and training, inadequate handling practices, and low usage of personal protective equipment [121]. Two studies conducted in Turkey found that only about 40% of participants used biological safety cabinets and that personal protective equipment was not used consistently [113,123]. Two studies conducted in Iran reported that antineoplastic drug handling practices were not always consistent with published recommendations [120,122].

Few studies have been conducted on the many chemical hazards in health care work. The only studies which could be found examined exposure to latex and antineoplastic drugs and there were no studies on other chemicals such as cleaning products, disinfectants and diathermy smoke.

Health care workers can also be exposed to physical hazards such as radiation, noise, and slips and falls [12]. However, this review did not identify any studies on exposure to these types of hazards from LMICs.

### 4.5. Implications

This scoping review has revealed that health care workers in LMICs are exposed to a wide range of occupational hazards and that risk reduction strategies and safety measures are inadequately implemented, mainly due to equipment and human resource limitations. To protect health care workers in these countries, first and foremost, occupational health and safety needs to be prioritised. This requires political commitment from governments to increase investments in occupational health and safety programs. Additionally, although development and public health agencies have promoted the importance of health care workers by including the health care workforce as an essential component of sustainable development, these agencies have focused mainly on increasing the numbers and competency of health care workers [138]. There is a need for these agencies to equally address the underlying reasons for health care workers’ migration, death and illness in LMICs and to advocate for the provision of safer workplaces for health care workers in these countries.

It is encouraging that research on occupational hazards among health care workers in LMICs has increased considerably in the past decade. However, the majority of the studies in this review were cross-sectional and some of them were of low quality (quality was not an exclusion criteria). In future, larger, more well-designed and prospective studies need to be conducted to make a convincing case for prioritising occupational health and safety of health care workers in these countries. In addition, the majority of the studies were on biological hazards and there were very few studies assessing exposure to chemical hazards. This is as expected since the risks from biological hazards are more apparent in LMICs where the population rates of infectious diseases are high. However, health care workers are also routinely exposed to chemicals that have been linked to chronic diseases such as cancer and asthma. More research is required in this area from LMICs.

### 4.6. Strengths and Limitations of the Review

To our knowledge, this review on exposure to occupational hazards among health care workers is the most comprehensive to date. It was based on a rigorous, systematic search strategy across five large databases with no date restrictions using strict methodological inclusion criteria.

Although this review has provided an overall synopsis of occupational hazards in health care workers in LMICs, there are some limitations to this study. First, the quality of the included studies was not assessed, so the review is inclusive of all articles irrespective of their quality. Second, only articles published in English were included, which might have resulted in the omission of data published in other languages. Thirdly, there is a possibility that all data may not have been captured by the search strategy, particularly if the articles were published in journals not indexed in Medline. Lastly, this review also excluded night shift work, which is an important occupational risk for health care workers. Despite these limitations, this review provides a comprehensive overview of the hazards encountered in the workplace by health care workers in LMICs.

## 5. Conclusions

Large proportions of health care workers in LMICs are occupationally exposed to a wide range of hazards. Safety measures and risk reduction strategies in these countries are suboptimal, mainly due to resource limitations. Health care workers need to be protected from occupational hazards because these hazards have the potential to cause diseases and injuries and can adversely impact the retention of health care workers and the quality of care provided. Health care worker retention is of particular importance in LMICs since these countries already face a shortage of health care workers. Political commitment towards making occupational health and safety a priority public health issue is necessary to ensure the safety of health care workers in LMICs. Although research on occupational hazards among health care workers in these countries has increased considerably in the last decade, most of this work is on biological hazards. More research is needed on the other types of occupational hazards.

## Figures and Tables

**Table 1:** The characteristics of the studies (n = 47) on exposure to biological hazards (arranged in chronological order according to the year of publication).

Authors	Year	Topic	Origin	Participants	Type of Study	Methods	Findings
**I. Bloodborne Pathogens**							
Cavalcante et al. [27]	1991	Occupational risk of acquiring HIV	Brazil	651 health care workers from a teaching hospital	Prospective	Health care workers who reported accidental exposures to infective material from AIDS patients to the Infection Control Committee (n = 247) and those who had other risks of infection but no occupational exposures (n = 404) were interviewed and blood was collected for HIV testing at baseline, 90, 180 and 360 days later (for health care workers who reported accidents).	247 health care workers reported 338 accidents and of these 115 were followed up for more than 6 months and 132 were lost to follow up. None tested positive for HIV. 50% of exposures occurred through needlestick or sharp injuries, 22% through contact of blood on mucous membranes, 28% through exposures to urine, faeces or cerebrospinal fluid from AIDs patients. The highest frequencies of injuries were reported by nurses, followed by physicians, laundry and housekeeping personnel and laboratory workers. Of the 404 health care workers with no occupational exposures, 6 were positive and had confirmed risk factors for HIV transmission.
Adegboye et al. [28]	1994	Needlestick and sharp object injuries and accidents; awareness of the risk of occupational exposure to HIV	Nigeria	474 health care workers working in a university hospital complex, who were occupationally exposed to blood	Cross-sectional	Questionnaires on needlestick and sharp injuries in the past year and on knowledge on HIV transmission.	27% of health care workers reported at least one needlestick injury in the past year. Circumstances resulting in needlestick injuries were unexpected patient movement (29%), handling or disposal of used needles (23%), needle recapping (18%), accidental stick by a colleague (18%), and needle disassembly (10%). 15% reported at least one sharp object injury in the past year and this most commonly involved broken glass from patient specimen containers (39%).The highest frequencies of injuries were reported by dental staff and surgeons. Almost all participants were aware of the risk of occupational exposure to HIV.
Olubuyide [29]	1996	Contact with HIV and Hepatitis B (HBV)-positive patients and needlestick injuries	Nigeria	149 resident doctors in a teaching hospital in Nigeria	Cross-sectional	Questionnaire asking about contact with HIV/HBV patients, needlestick injuries, and precautions used. No time period was reported.	93% reported contact with HIV/HBV patients, 9% had needlestick injuries (presumably lifetime) and 54% used universal precautions when performing procedures.
Gumodoka et al. [30]	1997	Injuries and use of personal protective equipment (PPE) to protect from HIV	United Republic of Tanzania	403 health care workers from nine hospitals in the Mwanza region	Mixed methods	Questionnaires on the use of PPE and needlestick injuries and splashes. Observations and interviews were carried out in different sections of the hospitals to determine general hygiene practices.	Prick and splash incidents were reported frequently (at least 5 pinprick accidents and nine splashes per health care worker per year). The general hygiene measures to reduce the risk of HIV was not sufficient and PPE was not used consistently.
Khuri-Bulos et al. [31]	1997	Needlestick and sharp injuries	Jordan	248 health care workers working in a tertiary care hospital	Prospective	Surveillance of sharps injuries over a 3 year period. Health care workers who reported sharps injuries during this period completed a questionnaire. Serum samples were collected at baseline and 6 months later to be tested for Hepatitis B, C and HIV.	Over the 3 year period, 248 health care workers reported needlestick injuries. Highest frequencies were reported by nurses (34.6%). The total average annual rate was 82:1000 health care workers per year. Only a minority of health care workers submitted a serum sample.
Gounden and Moodley [32]	2000	Injuries and use of personal protective equipment to protect from HIV	South Africa	265 health care workers from a tertiary care hospital	Mix of retrospective and prospective	Health care workers were interviewed over a period of one year.	13% reported accidental injuries with HIV-positive patients. The highest frequencies of injuries were among registrars. Compliance with universal precautions was suboptimal. 48% of the participants on post-exposure prophylaxis (PEP) did not complete their regimen; the side effects of PEP was reported as the main reason for discontinuation.
Phipps et al. [33]	2002	Needlestick injuries; and knowledge, attitudes and practices	China	441 nurses working in 3 tertiary care hospitals in Hunan Province	Cross-sectional	Questionnaire on needlestick injuries in the past year, Hepatitis B knowledge and immunization status, and work practices.	82% of the nurses reported experiencing a needlestick injury in the past year. These injuries occurred most frequently when separating a needle and syringe, recapping a needle, transporting needles for disposal, and giving injections. Only 8% reported the injuries to an authority. The majority never wore gloves when drawing blood, giving an injection or starting an intravenous line. 29% were not vaccinated against Hepatitis B.
Talaat et al. [34]	2003	Needlestick injuries and Hepatitis B vaccination status	Egypt	1485 health care workers from health facilities in 2 governorates (Nile Delta and Upper Egypt)	Cross-sectional	Questionnaire on needlestick injuries and Hepatitis B vaccination status.	36.6% reported at least one needlestick injury in the past 3 months. Two-hand recapping was the most common behaviour associated with needlestick injury. 15.8% reported being fully vaccinated against Hepatitis B; vaccination rates were lowest among housekeeping personnel.
Kermode et al. [35]	2005	Needlestick injuries	India	266 health care workers from 7 rural health settings (hospitals with attached community health projects) in north India	Cross-sectional	Questionnaire on needlestick or sharps injuries in the past week, past year, and over the working lifetime.	63% reported at least 1 needlestick injury in the last year and 73% over their working lifetime. Doctors and nurses were more likely to be exposed than student nurses and laboratory workers.
Kermode et al. [36]	2005	Compliance with universal precautions (UP)	India	266 health care workers from 7 rural health settings (hospitals with attached community health projects) in north India	Cross-sectional	Questionnaire on 12 behaviours related to the practice of UP.	Compliance with UP was not optimal. Compliance with UP was associated with being in the job for a longer period, knowledge of bloodborne pathogen transmission, perceiving fewer barriers to safe practice and strong commitment to workplace safety climate.
Nsubuga and Jaakkola [37]	2005	Needlestick and sharps injuries and risk factors	Uganda	526 midwives and nurses in a tertiary care hospital in Kampala	Cross-sectional	Questionnaire on needlestick injuries and risk factors.	57% reported a needlestick injury in the last year and 82% in their entire career. The risk factors identified were lack of training, working for more than 40 h/week, recapping needles, and not using gloves when handling needles. Lack of training was the strongest predictor.
Obi et al. [38]	2005	Needlestick injuries and splashes, and use of personal protective equipment (PPE)	Nigeria	264 surgeons from five tertiary health institutions in Southeast Nigeria	Cross-sectional	Questionnaire on needlestick injuries and splashes in the last 5 years, use of PPE, and attitudes towards care of HIV-infected patients.	40.2% reported a needlestick injury and 26% reported blood splashes in the past five years. The highest frequencies were reported in resident surgeons. All wore protective aprons, 65.2% used double gloves and 30.3% used goggles during surgical procedures. 83% had some reservations about treating HIV-positive patients.
Chelenyane and Endacott [39]	2006	Infection control practices	Botswana	22 health care workers from two referral hospitals emergency departments	Mixed methods	Questionnaire with multiple choice and open ended questions.	The majority of participants reported compliance with universal precautions. Barriers to compliance were lack of appropriate facilities, shortage of equipment and materials, inadequate staffing, and lack of training programs.
Akinleye and Omokhodion [40]	2008	Needlestick injuries and work practices	Nigeria	270 primary health care workers from two urban and three rural local government areas	Cross-sectional	Questionnaire on needlestick injuries in the past year and work practices.	32% reported a needlestick injury in the past year. Compliance with the use of gloves and hand washing were greater among rural than urban health workers.
Okeke et al. [41]	2008	Needlestick injuries and Hepatitis B vaccination status	Nigeria	346 medical students in a tertiary institute	Cross-sectional	Questionnaire on needlestick injuries and splashes, and Hepatitis B vaccination status	48% reported a previous needlestick injury and 27.7% reported being vaccinated against Hepatitis B.
Taegtmeyer et al. [42]	2008	Needlestick injuries (NSIs) and safety practices	Kenya	650 health care workers from 11 health facilities in Thika District	Prospective	Questionnaires and semi-structured interviews; together with an intervention of introduction of biosafety measures, vaccination, and post-exposure prophylaxis (PEP). Surveys were conducted at baseline and at one year.	The incidence of NSIs was 0.97 per health care worker per year. After the institution of biosafety measures, there was a significant reduction in injuries, an increase in the health care workers accessing HIV testing and in the uptake of Hepatitis B vaccination uptake, but the uptake of PEP was low.
Chen et al. [43]	2009	Sharp object injuries	China	831 health care workers from 9 hospitals in Fujian, who worked in departments with a high risk of occupational exposures to blood	Cross-sectional	Questionnaire on sharp object injuries in the past year.	86.2% of the health care workers reported a sharps injury on the job and 71.3% said that it had occurred in the past year. Nurses reported the highest frequencies of injuries, followed by surgeons, anaesthetists, and laboratory workers. Disposable syringes caused most of the injuries.
Simon [44]	2009	Needlestick injuries	India	50 nurses in a super-speciality tertiary care hospital in Delhi	Cross-sectional	Questionnaire on needlestick injuries, and knowledge and practices on needlestick injuries.	70% had sustained a needlestick injury during their career, and of these the majority (71%) did not report it. There was a lack of awareness on prevention and management of NSIs.
Chakravarthy et al. [45]	2010	Sharps injuries, and blood and body fluid exposure incidents	India	265 health care workers who reported sharps injuries and accidental blood and body fluid exposures to the Infection Control Committee of 4 tertiary referral hospitals	Retrospective review of data from sharp injury, and blood and body fluid exposure reports	Data were obtained from sharps injuries, and blood and body fluid exposures reports that were reported to the Infection Control Committees of the 4 hospitals. Data collection period ranged from 6 to 26 months.	243 sharps injuries and 22 incidents of blood and body fluids exposures were reported in the cumulated 50 months of study. The highest frequencies of injuries were reported by nurses and housekeeping staff. The majority of the injuries were caused by disposable needles.
Yacoub et al. [46]	2010	Needlestick injuries and Hepatitis B vaccination status	Syria	321 health care workers from three tertiary care hospitals in Aleppo	Cross-sectional	Questionnaire on needlestick injuries and Hepatitis B vaccination status. Blood was collected to test for Hepatitis B (HBsAg).	76.6% reported at least one needlestick injury in the past year. Anaesthesiology technicians, doctors, nurses, and housekeeping had the greatest exposure risks. 56.1% reported being fully vaccinated against Hepatitis B; vaccination rates were lowest among housekeeping personnel. 2.8% tested positive for HBsAg.
Sangwan et al. [47]	2011	Needlestick injuries and splashes	India	70 health care workers in a tertiary care hospital	Cross-sectional	Questionnaire on needlestick injuries and splashes in the past year, and reasons for not using PPE.	71.43% reported a needlestick injury in the past year. The most frequent reasons for not using PPE were in emergencies and other co-workers not using them. Only 34% reported that adequate PPE was always provided.
Irmak [48]	2012	Needlestick and sharps injuries	Turkey	143 nurses working patient care in a state hospital	Cross-sectional	Questionnaire on needlestick and sharps injuries in the past year.	30.1% of the nurses reported at least one sharp object injury in the past year. The use of syringe needles was the most common cause of injury. 16.3% of the nurses were not wearing gloves when they sustained the injury.
Nasim et al. [49]	2012	Safe work practices and use of personal protective equipment	Pakistan	1782 laboratory technicians from public sector hospitals and private hospital laboratories throughout Pakistan	Cross-sectional	Questionnaire on safe and unsafe work practices, and the use of personal protective equipment.	31.9% did not use any kind of personal protective equipment, 46% reported reusing syringes, 43.2% regularly recapped needles after use, 67.2% said that standard operating procedures were not available, and 84.2% had no formal biosafety training.
Omorogbe et al. [50]	2012	Injection safety practices and use of PPE	Nigeria	122 nurses from 6 mission hospitals in Benin city	Cross-sectional	Questionnaire adapted from the WHO injection safety assessment tool and observation of practices.	55.8% reported recapping of needles and only 3.3% said that they regularly used gloves when giving injections.
Phillips et al. [51]	2012	Needlestick and sharps injuries	Zambia	442 health care workers from five health facilities in Lusaka and Livingstone	Cross-sectional	Questionnaire on needlestick and sharps injuries in the past year.	The annual average sharps injury rate was 1.3 injuries/worker. The highest frequencies were reported by nurses and service workers. Syringe needles accounted for the majority of the injuries. 88% reported the availability of PPE, but only 8% were fully vaccinated against Hepatitis B.
Sethi et al. [52]	2012	Compliance with infection control practices	Uganda	183 health care workers from a referral hospital in Kampala	Cross-sectional	Questionnaire on hand hygiene, barrier protection, and contact precautions.	68.9% reported using gloves as barrier protection. Universal precautions were not always followed. The reasons for suboptimal infection control practices were lack of time and lack of resources.
Abkar et al. [53]	2013	Unsafe injection practices	Yemen	127 health care workers from two hospitals and 6 rural health centres	Cross-sectional	Questionnaire and observation of injection practices.	There were several unsafe practices, particularly the recapping of needles after use, which occurred in 61.1% and 36.8% of the observations in the hospitals and health centres, respectively.
Afridi et al. [54]	2013	Needlestick injuries, Hepatitis B vaccination status and infection control measures	Pakistan	497 health care workers from two tertiary care hospitals in Karachi	Cross-sectional	Questionnaire on needlestick injuries, Hepatitis B vaccination status and infection control measures.	64% reported needlestick injuries during their career. Working for more than 5 years and working as a nurse were the factors associated with an increased risks. Injecting medicine, drawing blood, and two hand recapping of needles were the practices associated with needlestick injuries. 34% reported being vaccinated against Hepatitis B. Infection control measures were inadequate.
Rajkumari et al. [55]	2014	Needlestick injuries and splashes	India	356 health care workers who reported sharps injuries and splashes in a tertiary hospital in New Delhi	Prospective	Surveillance of sharps injuries over a 2 years 5 months period. Health care workers who reported sharps injuries during this period completed a questionnaire. Blood samples were collected at baseline and 6 months later to be tested for Hepatitis B, C and HIV.	Highest frequencies of sharps injuries were reported by doctors (36.2%), followed by nurses (14.6%) and hospital waste disposal staff (7.6%). There was no seroconversion among the exposed health care workers. The majority (85.1%) of the injuries reported were from sharps (as compared to splashes). Only 55.3% were using PPE during the time of exposure.
Bekele et al. [56]	2015	Needlestick injury reporting and attitudes	Ethiopia	340 health care workers from four hospitals of Bale zone	Cross-sectional	Questionnaire on needlestick injury reporting and attitudes.	98.2% were aware of the risks of needlestick injuries.58.7% of needlestick injuries were not reported. The main reasons for not reporting were time constraints, sharps that caused the injury were not used by patients, the source patient did not have diseases of concern, and lack of knowledge of reporting.
Priya et al. [57]	2015	Needlestick injuries and splashes	India	105 health care workers who reported sharps injuries and accidental blood and body fluid exposures to the anti-retroviral therapy centre of a tertiary care hospital	Retrospective review of data from sharp injury, and blood and body fluid exposure reports	Data from three years were obtained from sharps injuries, and blood and body fluid exposures reports that were reported to the Anti-retroviral therapy centre of a tertiary care hospital.	105 health care workers reported an occupational exposure to blood and body fluids. The highest frequencies were reported by interns. Needlestick injuries were the commonest type of exposure (85%), followed by mucous membrane splash (13%) and exposure on intact skin (2%). The practices that resulted in exposures were blood withdrawal (45.7%), during surgical procedures (24.7%) and disposal of sharps (23%).
Sabermoghaddam et al. [58]	2015	Needlestick injuries and splashes	Iran	371 health care workers from 6 government hospitals in the Northern Khorasan province	Cross-sectional	Questionnaire on needlestick injuries and splashes in the past year.	44% reported a sharp object injury and 31% reported contact with blood and body fluids in the past year. 91% reported always using a safety box to deposit used needles, 35.9% reported washing their hands before and after examining patients, 41.5% reported using gloves, 58% had attended training on safe handling of sharps. 52% of those who were injured did not report the injury.
Türe et al. [59]	2016	Needlestick injuries and splashes, and risk factors	Turkey	166 health care workers who reported sharps injuries and accidental blood and body fluid exposures to the Infection Control Committee of a tertiary care hospital	Retrospective review of data from sharp injuries, and blood and body fluid exposure reports	Data were obtained from sharps injuries, and blood and body fluid exposure reports that were reported to the Infection Control Committee. Data collection period was from August 2011 to June 2013.	166 health care workers reported an occupational exposure to blood and body fluids. The occupational exposure incidence was 2.18 exposures/person-year. The highest frequencies of injuries were reported by nurses and cleaning staff. Having heavy workloads and long working hours increased the risk of exposures whereas increased work experience decreased the risk of exposures.
Konlan et al. [60]	2017	Hepatitis B vaccination status and practices to reduce occupational exposures	Ghana	108 nurses from two hospitals within the Tamale metropolis	Cross-sectional	Questionnaire on Hepatitis B vaccination status and practices to reduce occupational exposures to Hepatitis B.	64.8% said that they reported occupational exposures to Hepatitis B. 33.3% reported receiving 3 doses of Hepatitis B vaccination. Compliance with precautions to reduce occupational exposures was suboptimal.
Matsubara et al. [61]	2017	Needlestick and sharps injuries and risk factors	Lao PDR	623 health care workers from 4 tertiary care hospitals in Vientiane Capital	Cross-sectional	Questionnaire on needlestick injuries over their entire career, and in the past 6 months, and injection practices based on the World Health Organization questionnaire on injection practices.	11.4% reported a needlestick injury in the past 6 months and 42.1% in their entire career. The highest frequencies were reported by surgeons, dentists and cleaners. The injuries were caused by percutaneous injections (17.9%), suturing needles (17.0%), intravenous line insertion (17.0%), recapping needles (13.2%), disposal (10.4%), and others (24.5%). Protective factors for needlestick injuries identified were adequate availability of needles and syringes, and adequate training.
Geberemariyam et al. [62]	2018	Needlestick injures and infection control practices	Ethiopia	648 health care workers with direct involvement in patient care in public health care facilities in one district	Cross-sectional	Questionnaire on needlestick injuries and infection control practices.	Only 36.3% reported safe infection prevention practices. Life-time prevalence of needlesticks and blood or body fluid exposure 32.4% and 39.0%, respectively, with 24.8% of them having >1 injuries. Exposures occurred mostly during intravenous catheter insertion, suturing, and recapping of needles. Factors associated with better infection control practices were profession, service years, presence of infection prevention committee and guideline, and ever taking training.
Mandić et al. [63]	2018	Needlestick injuries and splashes	Serbia	5247 health care workers who routinely worked with blood from 17 general hospitals in Serbia	Cross-sectional	Questionnaire on needlestick injuries and splashes over their entire career and in the last year.	39% reported an exposure to blood and body fluids in the past year and 66% over their entire career. The prevalence of needlestick injuries occurring in the last year was equal among genders, but it was more prevalent in women during the entire career. The highest frequencies were reported in nurses.
Hebo et al. [64]	2019	Exposure to blood and body fluids, practices of standard precautions and seroprevalance of Hepatitis B and C	Ethiopia	240 health care workers from Jimma University Medical Center	Cross-sectional	Questionnaires on exposure to blood and body fluids and use of standard precautions. Blood was collected and tested for Hepatitis B and C.	60% reported being ever exposed and 43% reported exposure in the past year to blood and body fluids through accidental splashes and sharps injuries. 2.5% of the samples was positive for HBsAg and 0.42% for anti-HCV antibodies. Only 42.6% had good practices of standard precautions.
**II. Tuberculosis (TB)**							
Lien et al. [65]	2009	Prevalence of latent TB and risk factors	Vietnam	150 health care workers from a TB hospital and 150 from a non-TB hospital in Hanoi	Cross-sectional	Questionnaire on occupational history; interferon-gamma release assay (IGRA), QuantiFERON-TB Gold In-Tube assay and one- and two-step tuberculin skin tests (TSTs) for TB infection.	Prevalence of TB infection was 47.3%, 61.1% and 66.3% as estimated by IGRA, one- and two-step TST, respectively. Working in a TB hospital, increasing age, lower education levels, and higher body mass index were associated with increased risk of IGRA positivity.
Mathew et al. [66]	2013	TB among health care workers	India	101 health care workers with TB (cases) and 101 without TB in a tertiary care hospital in Vellore	Nested case–control	Questionnaire on occupational history and non-occupational exposure to TB.	Rate of active pulmonary TB was 314 per 100,000 person-years, which was 1.86 times higher than that of the general population. Body mass index <19 kg/m^2^, having frequent contact with patients, and working in the medical wards or microbiology laboratories were independently associated with increased risk of TB
Wei et al. [67]	2013	Prevalence of latent TB infection (LTBI)	China	210 health care workers in a chest hospital in Harbin	Prospective	Questionnaire on occupational history; participants were tested with two interferon-gamma release assays (QuantiFERON-TB Gold In-Tube assay (GFT-GIT) and A.TB) and TST. Participants were observed for 2 years to check for the development of active tuberculosis.	Prevalence of LTBI was 76.5% by QFT-GIT, 65.7% by A.TB and 97.6% by TST, which was higher than that reported in the general population. Working as a nurse and age > 30 years were independently associated with increased risk of LTBI.
Whitaker et al. [68]	2013	Prevalence and risk of latent TB infection (LTBI)	Georgia	319 health care workers in Georgia	Prospective longitudinal	Questionnaire, and tests for LTBI using the TST and QuantiFERON-TB Gold In-Tube Assay (QFT-GIT). The tests were repeated 6–26 months after baseline.	Prevalence at baseline was 67% by TST and 46% by QFT-GIT. Health care workers (HCWs) working in TB health care facilities had a higher prevalence of positive TST and QTF-GIT. Frequent contact with TB patients was associated with increased risk of QTF-GIT positivity only and increasing age was associated with increased risk of positivity of both tests. The conversions rates were high at 22.8/100 person-years (QTF-GIT) and 17.1/100 person-years (TST). Female HCWs had a decreased risk of TST conversion and older HCWs had an increased risk of QTF-GIT conversion.
Tudor et al. [69]	2014	TB incidence and risk factors	South Africa	1313 health care workers from 3 district hospitals in KwaZulu-Natal	Retrospective	Occupational health medical records of 1313 health care workers were reviewed during the period of January 2006 and December 2010.	The TB incidence rate was 1958/100,000 person-years, which was two-fold greater than in the general population. An increased incidence of TB was seen in those working in TB wards, paediatric wards, outpatient departments and stores/workshops. Health care workers living with HIV had a greater incidence of TB.
El-Sokkary et al. [70]	2015	Latent TB infection (LTBI) prevalence and risk factors	Egypt	132 health care workers from a chest Hospital in Zagazig city	Cross-sectional	Questionnaire and tests for LTBI using the TST and QuantiFERON-TB Gold In-Tube Assay (QFT-GIT).	Prevalence was 28.8% by QFT-GIT and 59.1% by TST. Being a nurse, working >10 years, smoking and diabetes were significantly associated with risk of LTBI.
Tudor et al. [71]	2016	Occupational risk factors for TB	South Africa	145 health care workers (54 cases, 91 controls) from 3 district hospitals in KwaZulu-Natal	Case control	Cases were identified from the occupational health medical records between January 2006 and December 2010.	Health care workers with HIV and those who spent time working in areas with tuberculosis patients were at an increased risk of TB.
He et al. [72]	2017	Pulmonary tuberculosis status among health care workers as diagnosed with low-dose CT	China	1012 health care workers from the Beijing Chest Hospital	Retrospective	Health examination data of 1012 health care workers which included low-dose lung CT examinations from January 2012 to November 2015 were analysed.	The incidence and prevalence rates of active TB were >2.8 times and >4.1 times greater than that of the general population of China. The majority (78.9%) of the health care workers with active TB worked in high-risk areas such as TB wards, outpatient clinics and radiology departments.
Erawati and Andriany [73]	2020	Latent TB infection (LTBI) prevalence and risk factors	Indonesia	195 health care workers from 34 primary health centres in Semarang	Cross-sectional	Questionnaire and tests for LTBI using QuantiFERON-TB Gold In-Tube Assay (QFT-GIT).	Prevalence of LTBI was 23.6%. Health care workers with comorbidities were at increased risk of LTBI.

Notes: HIV—Human Immunodeficiency Virus, AIDS—Acquired Immunodeficiency Syndrome, HBV—Hepatitis B Virus, HCV—Hepatitis C Virus, PPE—personal protective equipment, PEP—post-exposure prophylaxis, UP—universal precaution, NSI—needlestick injury, HBsAg—Hepatitis B surface Antigen, TB—tuberculosis, IGRA—interferon-gamma release assay, TST—tuberculin skin test, LTBI—latent tuberculosis infection. Mixed-methods studies refers to studies with qualitative and quantitative components.

**Table 2:** The characteristics of the studies (n = 22) on exposure to psychosocial hazards (arranged in chronological order according to the year of publication).

Authors	Year	Topic	Origin	Participants	Type of Study	Methods	Findings
**I. Workplace Violence**							
Kisa et al. [74]	2002	Sexual harassment and work productivity	Turkey	215 nurses from two hospitals in Turkey	Cross-sectional	Questionnaires on sexual harassment and work performance.	73% reported being sexually harassed. The main perpetrators were physicians and patients, and these incidents occurred more commonly in the in-patient clinics. 45% reported a decline in work productivity following the incidents.
Kamchuchat et al. [75]	2008	Workplace violence	Thailand	545 nurses working in a general hospital in southern Thailand	Mixed methods	Questionnaire modified from one developed by the Joint Program on Workplace Violence in the Health Sector and key informant interviews (n = 17).	The 12-month prevalence was 38.9% for verbal abuse, 3.1% for physical abuse and 0.7% for sexual harassment. The main perpetrators of verbal and physical abuse were patients and their family, while co-workers were the main perpetrators for sexual harassment. Younger age and working in high-risk areas (out-patient unit, emergency units, operating theatre, medical and surgical units) were associated with an increased risk of violence.
Aydin et al. [76]	2009	Workplace violence	Turkey	522 general practitioners from 48 cities	Cross-sectional	Questionnaire on workplace violence.	82.2% reported experiencing violence at work. Verbal abuse was the most common (89.3%), followed by physical violence (7.9%), economic (1.7%) and sexual violence (1.1%). Verbal and sexual violence was more common in women and physical and economic violence more common in men. Patients and their relatives was the most common source (91.1%).
Gimeno et al. [77]	2010	Prevalence of verbal abuse and its association with safety climate at work	Costa Rica	625 health care workers working in 10 public hospitals in Costa Rica	Cross-sectional	Questionnaires on safety climate and verbal abuse.	83.9% of the participants reported low safety climate levels. Prevalence of verbal abuse from all sources was 78.2%, with the most common being abuse from co-workers and patients. The odds of experiencing verbal abused increased with lower levels of safety climate.
Atan et al. [78]	2012	Workplace violence	Turkey	441 nurses from 6 university hospitals	Cross-sectional	Questionnaires on workplace violence in the past year.	60.8% reported some form of workplace violence, 59.4% verbal violence and 16.6% physical violence. The sources for verbal violence were patients (47.4%), visitors (39.5%), and health staff (10.7%) and for physical violence were patients (14.3%), visitors (5.0%) and health staff (0.5%). Of those who experienced violence, 42.9% reported a negative impact on their physical and/or psychological health and 42.9% reported a negative impact on work performance.
Khademloo et al. [79]	2013	Prevalence of physical and verbal abuse	Iran	271 nurses from 5 hospitals in the north of Iran	Cross-sectional	Questionnaire on physical and verbal abuse experienced in the last year (Staff Observation Scale Revised (SOAS-R)).	95.9% reported verbal abuse; the sources were patients (30.3%), family members (53.4%), and co-workers (16.1%). 29.1% reported physical abuse; the sources were patients (44.3%) and family members (55.6%).
da Silva et al. [80]	2015	Workplace violence and its association with depression	Brazil	2940 primary health care workers from 66 health centres in Sao Paolo	Cross-sectional	Questionnaire on workplace violence (adapted from a WHO questionnaire on domestic violence), and depression and depressive symptoms (Brazilian version of the nine-item Patient Health Questionnaire).	The frequencies of violence experienced at work were: insults (44.9%), witnessing violence (29.5%), threats (24.8%), and physical aggression (2.3%). Exposure to violence was positively associated with depressive symptoms and probable major depression.
Baig et al. [81]	2018	Prevalence of workplace violence	Pakistan	822 health care workers from hospitals, non-government organizations and ambulance services in Karachi	Mixed methods	Questionnaires on workplace violence; and 42 in-depth interviews and 17 focus group discussions.	33.5% had experienced violence in the past year. Verbal violence was more common (30.5%) than physical violence (14.6%). The main source was from people who accompanied patients (58.1%). The main perceived causes of violence were failure to meet the expectations of patients, communication gaps, poor quality of services, inadequate security in facilities, heavy workloads, and lack of training to respond to violence.
Zhao et al. [82]	2018	Prevalence of workplace violence and association with mental health	China	886 nurses from 8 tertiary hospitals in Heilongjiang Province	Cross-sectional	Questionnaires on workplace violence (Workplace Violence Scale), anxiety (Self-rating Anxiety Scale) and depression (Self-rating Depression Scale).	67.2% reported workplace violence. Workplace violence was positively associated with anxiety and depression. Service years played a moderating role in the relationship between workplace violence and anxiety, and gender played a moderating role in the association between workplace violence and depression.
Abate et al. [83]	2019	Workplace violence and associated factors	Ethiopia	435 health care workers from a tertiary care mental hospital in Addis Ababa	Cross-sectional	ILO/ICN/WHO/PSI Workplace Violence in the Health Sector Country Case Study Questionnaire.	62.1% reported verbal violence, 36.8% physical violence and 21.8% sexual harassment. Age > 31 years and contact with patients were the associated factors.
Yenealem et al. [84]	2019	Prevalence and risk factors for violence at work	Ethiopia	531 health care workers from Gondar city	Cross-sectional	Questionnaires adapted from the ILO/ICN/WHO/PSI Workplace Violence in the Health Sector Country Case Study Questionnaire.	58.2% reported experiencing some form of violence, of which 53.1% reported verbal abuse, 22% physical attacks, and 7.2% sexual harassment. Working in emergency departments, working in shifts, having less work experience and being a nurse was associated with an increased risk of violence.
Hacer and Ali [85]	2020	Workplace violence and its association with burnout	Turkey	310 physicians from Ordu province	Cross-sectional	Questionnaires on workplace violence and the Maslach Burnout Inventory.	93.2% reported experiencing verbal violence, 86.1% psychological violence and 22.6% physical violence. The most common source of violence were patients and their relatives. Emotional exhaustion and depersonalization scores were significantly higher in those who had experienced violence.
**II. Burnout**							
Ashkar et al. [86]	2009	Prevalence of burnout	Lebanon	155 resident doctors from 2 tertiary care hospitals in Beirut	Cross-sectional	Questionnaires on occupational history and the Maslach Burnout Inventory for Health Service Workers.	80% reported high levels of burnout in at least one domain. Prevalence according to subscales was: high levels of emotional exhaustion (EE)—67.7%, high depersonalisation (DP) scores—47.1% and low levels of personal accomplishment (PA)—23.9%. Working > 80 h/week, experiencing a major stress, getting > eight calls per month, and being female increased the risk of burnout
Ayala and Carnero [87]	2013	Demographic and occupational determinants of burnout	Peru	93 nurses working in acute and critical care departments in a referral military hospital in Lima	Cross-sectional	Questionnaires on occupational history and the Maslach Burnout Inventory.	Higher emotional exhaustion scores were associated with having children and inversely associated with time working in the current department. Higher depersonalisation scores were associated with being single and working in the emergency room or intensive care unit. Higher personal achievement scores were associated with having children.
Zubairi and Noordin [88]	2016	Prevalence of burnout and risk factors	Pakistan	82 resident doctors working in a university hospital in Karachi	Cross-sectional	Questionnaires on occupational history and the Maslach Burnout Inventory	74.4% reported high levels of burnout on at least one subscale, and 12.2% reported burnout on all the three subscales. Prevalence according to subscales was: high levels of EE—60%, high DP scores—38% and low levels of PA—32%. Workload dissatisfaction, length of working hours, relationship with co-workers and lack of autonomy were associated with an increased risk of burnout
Colindres et al. [89]	2018	Association of psychosocial work environment, burnout and compliance with infection control measures	Ecuador	333 nurses in four acute care facilities in Ecuador	Cross-sectional	Questionnaires on effort-reward imbalance, burnout (Copenhagen Burnout Inventory scale) and infection control compliance (modified Johns Hopkins University. School of Hygiene and Public Health Safety Climate Questionnaire).	21% of nurses experienced effort reward imbalance and 35.8% had work-related burnout. 44.2% reported adhering to infection control practices. Increased effort-reward imbalance was associated with an increased risk of burnout. Burnout was independently associated with decreased adherence to infection control practices.
Khan et al. [90]	2019	Job stress and burnout	Pakistan	447 anaesthesiologists from tertiary hospitals in Lahore and Karachi	Cross-sectional	Questionnaires on occupational history and the Maslach Burnout Inventory.	39.4% showed moderate to high levels emotional exhaustion, 68.4% moderate to high levels of depersonalization, and 50.3% moderate to high levels of burnout in personal achievements. Working in Lahore, > 2 nights on call per week, and > 40 h/week work inside the operating room were associated with burnout.
Mumbwe et al. [91]	2020	Prevalence of burnout	Zambia	160 anaesthesia providers (physicians and non-physicians) in Zambia	Cross-sectional	Questionnaires on occupational history and the Maslach Burnout Inventory.	Burnout was seen in 51.3% of participants. Prevalence according to subscales was: high levels of EE—66.3%, high DP scores—45% and low levels of PA—23.8%. Not being a physician and not having the right team to work with were significantly associated with burnout.
**III. Work Environment and Job Satisfaction**							
Li et al. [92]	2009	Psychosocial work environment and intention to leave	China	3088 nurses from 12 hospitals participated in the baseline study and 1521 in the one-year follow-up study	Longitudinal	Copenhagen Psychosocial Questionnaires.	Prevalence of intention to leave was 16.26% at baseline, and at one-year follow up, the incidence rate was 14.46%. Increased emotional demand, decreased workplace commitment, decreased meaning of work, and decreased job satisfaction were associated with intention to leave.
Ayamolowo et al. [93]	2013	Work environment and job satisfaction	Nigeria	161 nurses working in public primary health care facilities in Ekiti State	Cross-sectional	Questionnaires assessing work environment (adapted from the World Health Professions Alliance checklist on environment for health care professionals) and job satisfaction (Minnesota Satisfaction Questionnaire (MSQ).	44% of the nurses perceived their work environment to be of average quality and 31% as high quality. A majority (67.1%) of nurses reported low degrees of job satisfaction. There was a positive correlation between overall work environment and job satisfaction.
Ogunlade and Ogunfowokan. [94]	2014	Nurses’ experiences and satisfaction with night shift work	Nigeria	186 nurses who did a roster including night shift in 2 tertiary hospitals in Ile-Ife	Cross-sectional	Questionnaires assessing experiences and satisfaction during night shift work.	Overall, 55.4% were fairly satisfied with their night shifts as compared to 1.6% who were very satisfied and 43.0% who were satisfied. Inadequate staffing and equipment for protection from hazards were the factors that contributed to the low satisfaction with night shifts.
Ayalew and Workineh [95]	2019	Job satisfaction and associated factors	Ethiopia	220 nurses from public health facilities in Bahir Dar city	Cross-sectional	Questionnaire on job satisfaction using the Job satisfaction scale and Minnesota Questionnaire.	43.6% were satisfied with their job. Advancement, recognition and work security were positively associated with job satisfaction.

Note: Mixed-methods studies refers to studies with qualitative and quantitative components.

**Table 3:** The characteristics of the studies (n = 17) on exposure to ergonomic hazards (arranged in chronological order according to the year of publication).

Authors	Year	Topic	Origin	Participants	Type of Study	Methods	Findings
Smith et al. [96]	2004	Musculoskeletal complaints (MSCs) and psychosocial risk factors	China	282 nurses from a tertiary care hospital in Shijiazhuang city	Cross-sectional	Standardized Nordic Questionnaire.	Prevalence of MSCs in the past 12 months was 70%. The most common site was the lower back (56%) followed by the neck (45%), shoulder (40%) and upper back (37%). High mental pressure, limited work support and performing boring and tedious tasks were associated with increased risk of MSCs.
Tezel [97]	2005	Musculoskeletal complaints (MSCs)	Turkey	120 nurses from 4 hospitals in Ezrurum	Cross-sectional	Standardized Nordic Questionnaire.	90% reported at least one MSC in the past 6 months. Low-back pain was the most common (69%), followed by neck (54%) and shoulder (46%) pain.
Fabunmi et al. [98]	2008	Prevalence of musculoskeletal disorders (MSD)	Nigeria	214 nurses in a university hospital in Ibadan	Cross-sectional	Standardized Nordic Questionnaire.	90.7% reported experiencing MSDs in the past 12 months. Low-back pain was the most common (78%). Job inexperience, volume and type of work were the predisposing factors.
de Castro et al. [99]	2009	Work-related injuries and back pain	Philippines	690 nurses from 13 regions of the Philippines who were attending the Philippines Nurses Association annual national convention	Cross-sectional	Questionnaires on work related injuries/illness, reporting behaviour, and safety concerns.	38.6% reported experiencing at least one occupational injury/illness in the past year and 78.2% reported experiencing back pain. Most of the injuries were not reported. The most frequent safety concerns reported were stress and overwork.
Karahan et al. [100]	2009	Prevalence of low-back pain and risk factors	Turkey	1600 health care workers from 6 hospitals in 4 Turkish cities	Cross-sectional	Questionnaires on back pain and occupational history.	61.3% reported at least one occurrence of low-back pain within the last 12 months. Age, female gender, smoking, occupation as a nurse, work stress and heavy lifting were associated with increased risks.
Mehrdad et al. [101]	2010	Musculoskeletal symptoms and association with psychosocial factors	Iran	317 nurses from the Emam hospital in Tehran	Cross-sectional	Standardized Nordic Questionnaire and General Nordic questionnaire on psychosocial work environment.	95% reported complaints in at least one body site in the past 12 months. Low back was the most common site (73.2%). Higher levels of stress was associated with increased risk of musculoskeletal complaints.
Tinubu et al. [102]	2010	Work-related musculoskeletal disorders (WMSDs) and risk factors	Nigeria	128 nurses from 3 hospitals in Ibidan	Cross-sectional	Standardized Nordic Questionnaire.	78% reported WMSDs in at least one body site in the past 12 months. WMSDs occurred mostly in low back (44.1%), neck (28.0%), and knees (22.4%). Working in the same position for long periods, lifting/transferring patients, bending or twisting, and handling many patients were the commonest risk factors.
Arsalani et al. [103]	2014	Prevalence of musculoskeletal disorders (MSD) and risk factors	Iran	520 nurses working in 10 university hospitals in Tehran	Cross-sectional	Standardized Nordic Questionnaire and psychosocial working conditions from the Copenhagen Psychosocial Questionnaire.	88% reported experiencing MSDs in the past 12 months, with the most common body regions being the lower back (65.3%), knees (56.2%) and neck (49.8%). Physical and psychosocial work demands and low control over their work, which lead to work-related stress, increased the risk of MSDs. Participants also reported inflexible work schedule, poor quality of devices for transferring patients, overexertion and job dissatisfaction.
Barzideh et al. [104]	2014	Prevalence of musculoskeletal disorders (MSD) and risk factors	Iran	385 nurses working in 14 educational hospitals	Cross-sectional	Standardized Nordic questionnaire and Job Content Questionnaire.	89.9% reported experiencing MSDs in the last 12 months. Lower back pain was the most common (61.8%). High psychological and physical job demands and low decision latitude were associated with increased risks.
Munabi et al. [105]	2014	Prevalence of musculoskeletal disorders (MSD) and risk factors	Uganda	741 nurses from 5 hospitals in Uganda	Cross-sectional	Questionnaire adapted from the Standardized Nordic and standardized Dutch Musculoskeletal questionnaires.	80.8% had experienced MSDs in the last 12 months. Low-back pain was the most common (61.9%). Working in a bent or twisted position, mental exhaustion and being absent from work for more than 6 months were associated with an increased risk.
Yasobant and Rajkumar [106]	2014	Work-related musculoskeletal disorders (WMSDs), and risk factors	India	140 health care workers from a tertiary care hospital in Chennai	Cross-sectional	Standardized Nordic Musculoskeletal Questionnaire.	50.7% reported symptoms in at least one body site in the past 12 months. Low back was the most common site (45.7%). Working in the same position for long periods, working in awkward and cramped positions, and performing repetitive tasks were the commonest risk factors.
Abaraogu et al. [107]	2017	Work-related musculoskeletal disorders (WMSDs) and job stress	Nigeria	126 physiotherapists from hospitals in five states	Cross-sectional	Standardized Nordic Musculoskeletal Questionnaire and Job Content Questionnaire.	82.1% reported symptoms in at least one body site in the last 12 months. Low back was the most common site (57.8%). There were high levels of stress in most of the job dimensions. However, no specific domains of job stress dimensions were associated with WMSDs.
Amin et al. [108]	2018	Prevalence of self-perceived emotional distress and musculoskeletal disorders (MSD)	Malaysia	376 nurses working in public hospitals in the Klang valley	Cross-sectional	Standardized Nordic Musculoskeletal Questionnaire and short version of the Depression, Anxiety, and Stress Scale.	73.1% had experienced MSDs in the last 12 months and neck was the most common site (48.9%). 75% reported emotional distress. Stress and anxiety were significantly associated with an increased risk of MSDs.
Dlungwane et al. [109]	2018	Low-back pain and risk factors	South Africa	242 nurses from a regional hospital in KwaZulu-Natal	Cross-sectional	Questionnaire on back pain and risk factors.	The point prevalence of low-back pain was 59%. Frequent bending, maintaining prolonged positions and transferring patients were the risk factors.
Ike and Olawumi [110]	2018	Back pain and risk factors	Nigeria	228 nurses working in a medical centre in Abeokuta	Cross-sectional	Questionnaire on back pain and risk factors.	The point prevalence of back pain was 39%. Maintaining a particular position for long periods and lifting patients were common risk factors.
Luan et al. [111]	2018	Prevalence of musculoskeletal disorders (MSD) and risk factors	Vietnam	1179 nurses working in 15 district hospitals in Haiphong	Cross-sectional	Standardized Nordic Questionnaire.	74.7% reported symptoms of MSDs in the last 12 months. Low back and neck were the most common sites (44.4% and 44.1%). Age, history of musculoskeletal disease, anxiety and absenteeism in the workplace were risk factors.
Dong et al. [112]	2019	Prevalence of musculoskeletal disorders (MSD) and risk factors	China	14,720 health care workers from 8 tertiary hospitals in Shandong Province	Cross-sectional	Questionnaire incorporating the Standardized Nordic Musculoskeletal and the Dutch Musculoskeletal Questionnaires.	91.2% reported symptoms in at least one body site in the last 12 months. Low back was the most common site (72.8%). MSDs were associated with increased work load, psychological fatigue, mental stress and certain ergonomic factors (bending, twisting).

**Table 4:** The characteristics of the studies (n = 11) on exposure to chemical hazards and occupational hazards in general (n = 2) (arranged in chronological order according to the year of publication).

Authors	Year	Topic	Origin	Participants	Type of Study	Methods	Findings
**I. Chemical Hazards**							
Baykal et al. [113]	2009	Working conditions and safe handling practices of antineoplastic drugs	Turkey	171 nurses who worked in oncology units and administered antineoplastic drugs in nine hospitals in Istanbul	Cross-sectional	Questionnaires on working conditions and safe handling practices of antineoplastic drugs were distributed.	94.7% of the nurses reported wearing gloves, 89.5% wore masks, 52.0% wore gowns and 18.7% wore goggles. 40.4% reported preparing drugs in a biological safety cabinet, 37.4% said that they prepared the drugs in the nurses’ office and 15.8% said that they prepared the drugs in a room that was also used for other purposes such as meals.
Agrawal et al. [114]	2010	Exposure to latex and latex allergy	India	163 dental professionals working in Udaipur city	Cross-sectional	Questionnaires on latex glove use and symptoms of latex allergy.	16% reported allergy symptoms to latex gloves. 81.6% wore gloves for >5 h a day. The number of years of latex gloves use was significantly associated with allergic symptoms.
Amarasekera et al. [115]	2010	Exposure to latex and latex allergy	Sri-Lanka	325 health care workers in a tertiary care hospital	Cross-sectional	Questionnaires latex gloves use and symptoms of latex allergy.	16.3% reported latex allergy symptoms. 49.2% wore gloves for >1 h a day and 44.2% handled other rubber products at work. Longer duration of working as a health care worker and using gloves for >1 h/day were the risk factors associated with allergic symptoms.
Phaswana and Naidoo [116]	2013	Prevalence of latex sensitization and allergy with the use of hypoallergenic powder and lightly powdered latex gloves	South Africa	501 health care workers (337 who used latex gloves and 164 administration staff who did not use latex gloves) in a tertiary care hospital in KwaZulu-Natal	Cross-sectional	Questionnaires on latex glove use and symptoms of latex allergy. Skin prick tests were conducted for latex sensitization.	Prevalence of latex sensitisation and allergy in exposed workers was 7.1% and 5.9%, respectively; and in unexposed workers it was 3.1% and 1.8%. Work-related allergy symptoms were significantly higher in exposed workers. A dose-response relationship was observed for powdered latex gloves.
Supapvanich et al. [117]	2013	Exposure to latex and latex allergy	Thailand	899 nurses from three hospitals in Thailand	Cross-sectional	Questionnaires on respiratory and dermal symptoms that were attributed to latex gloves use.	18% reported symptoms attributable to latex gloves use. Dermal symptoms were more frequently reported, particularly itchy skin and rash. Using >15 pairs of powdered latex gloves/day, using chlorhexidine and being an operating theatre nurse were the risk factors associated dermal symptoms.
Köse et al. [118]	2014	Exposure to latex and latex sensitization	Turkey	1115 health care workers from an education and research hospital in Izmir	Cross-sectional	Questionnaires on latex gloves use and symptoms of latex allergy. Blood was tested for latex-specific IgE levels.	Prevalence of latex sensitization was 4.2%. Latex allergy was more common in nurses.
Supapvanich et al. [119]	2014	Exposure to latex and latex sensitization	Thailand	363 nurses from two tertiary hospitals in Southern Thailand	Cross-sectional	Questionnaires on use of latex gloves and symptoms related to latex use. Latex sensitization was confirmed by detecting anti-latex IgE antibodies using a solid phase immunoassay.	The prevalence of latex sensitization was 4.4%. The prevalence of latex sensitization was higher in hospitals where gloves with higher protein levels were used.
Abbasi et al. [120]	2016	Safe handling practices of antineoplastic drugs	Iran	86 nurses who worked in oncology units and administered antineoplastic drugs from six centres of chemotherapy in Shiraz	Cross-sectional	Questionnaires on the safe handling practices were distributed. Observation of work practices was performed using a check list.	Only about half of the nurses used personal protective equipment (PPE) during the administration of the drugs, and only about 5% used PPE during the administration and disposal of the drugs. Biological safety cabinets were used in all the hospitals and clinics included in the study.
Elshaer [121]	2017	Adherence to control measures used for handling of antineoplastic drugs	Egypt	54 nurses and clinical pharmacists who were exposed to ADs and 54 who were not exposed, working in oncology centres in Alexandria city.	Cross-sectional	Questionnaires on adverse health effects and control measures were distributed. Nurses and clinical pharmacists who were exposed to ADs were compared to those who were not exposed.	Biological safety cabinets and ventilation devices were used by pharmacists but not by nurses. Significantly higher percentages of pharmacists reported safe handling practices and the use of PPE as compared to nurses. There was no medical surveillance program in the workplace.
Alehashem and Baniasadi [122]	2018	Safe-handling practices of antineoplastic drugs and control measures	Iran	14 oncology health care workers filled 224 questionnaires in a tertiary care centre	Cross-sectional	7–8 health care workers worked in the Oncology ward every day. They filled the questionnaire on safe handling practices for six weeks or 30 working days.	20.56% reported carrying out drug preparations without any personal protective equipment. All preparations of antineoplastic drugs were reported to be performed in a biological safety cabinet.
Bayraktar-Ekincioglu et al. [123]	2018	Practices and safety measures when handling antineoplastic drugs	Turkey	40 hospital pharmacists who handled chemotherapy from Turkey	Cross-sectional	Questionnaires on chemotherapy drug preparation processes and knowledge on the safety measures.	The majority (42.5%) reported using automated chemotherapy units and 30% prepared the drugs manually. The reported practices were not always consistent with published recommendations: use of double glove (63.6%), glasses (62.2%), hair cap (66.7%), foot covers (32.3%), masks (89.1%), coat (92.1%), closed-system drug transfer set (70.6%), and biological safety cabinet (91.7%).
**II. Occupational Hazards (General)**							
Aluko et al. [124]	2016	Compliance with control measures	Nigeria	290 health care workers in Osun state	Cross-sectional	Questionnaires on knowledge on occupational hazards and their control practices.	Participants were knowledgeable about the various types of occupational hazards (biological, chemical, physical, and ergonomic). Regarding control practices, 96.2% wore gloves and 77.2 practiced correct body posturing during clinical procedures, 93.8% reported safe disposals, and 62.4% were immunized against Hepatitis B. Only 52.1% always complied with standard procedures and the main reasons for non-compliance were lack of safety equipment and time constrains.
Tait et al. [125]	2018	Biological, chemical, and physical hazards in medical laboratories	Kenya	204 laboratory workers in 108 medical laboratories in Kajiado county	Cross-sectional	Questionnaires on biological, chemical and physical hazards.	65.6% were exposed to 1 + biological hazard, 38.2% handled un-labelled and un-marked chemicals; and 49.5% reported laboratory equipment dangerously placed. There were a large number of other risks. Strong correlations between protective measures within individuals. Control measures reported were occupational health and safety training and supervising staff (98%), proper medical waste containers (92.6%), first aid safety equipment (36.8%), chemical hygiene plans (25%) and chemical hoods (19.1%).

Note: PPE—personal protective equipment.

## Data Availability

All data are presented in this article. Researchers can contact authors regarding any request about the data.

## Supplementary Materials

The following are available online at https://www.mdpi.com/1660-4601/18/5/2603/s1, Table S1: Search strategy for Medline (Ovid) (date of search: 1 May 2020).
