# Supplementary material for: Exposure to Occupational Hazards among Health Care Workers in Low- and Middle-Income Countries: A Scoping Review
Source: Int J Environ Res Public Health. 2021 Mar 5;18(5):2603. doi: 10.3390/ijerph18052603 (PMC7967386; doi:10.3390/ijerph18052603)
Supplement: Supplementary file 1 [file ijerph-18-02603-s001.pdf]

Exposure to occupational hazards among healthcare workers in low- and middle-income countries: A scoping review

Supplementary Table. Search strategy for Medline (Ovid) (date of search: 01/05/2020)

#	Search terms	Results
1	Health personnel/ or allied health personnel/ or dental staff/ or dentists/ or medical laboratory personnel/ or medical staff/ or nurses/ or nursing staff/ or personnel, hospital/ or pharmacists/ or physical therapists/ or physicians/	247759
2	Healthcare worker.mp. or exp Health Personnel/	508882
3	Exp Health Personnel/	508239
4	Doctors.mp.	79077
5	Health worker.mp.	3624
6	Exp Nurses/	87604
7	Laboratory workers.mp.	872
8	Exp Developing Countries/	74246
9	Developing Countries/ or low income countries.mp.	78538
10	Developing Countries/ or middle income countries.mp.	86404
11	Low and middle income countries.mp.	15851
12	1 or 2 or 3 or 4 or 5 or 6 or 7	566760
13	8 or 9 or 10 or 11	90218
14	12 and 13	5552
15	Exp Occupational Health/	32933
16	Occupational Health/ or Workplace/ or Occupational Diseases/ or occupational hazards.mp. or Burnout, Professional/ or Occupational Exposure/	174709
17	Occupational Health/ or Occupational Diseases/ or occupational risks.mp. or Occupational Exposure/	150603
18	Exp Occupational Diseases/	130883
19	Accidents, Occupational/ or Occupational Diseases/ or Occupational Injuries/	99729
20	Occupational accidents.mp. or Accidents, Occupational/	17686
21	Occupation.mp. or Occupations/	49467
22	Workplace Violence/ or Workplace/ or workplace.mp.	48977
23	15 or 16 or 17 or 18 or 19 or 20 or 21 or 22	276420
24	14 and 23	173
25	Limit 24 to English language	170
